# Metabolomic Response of Skeletal Muscle to Aerobic Exercise Training in Insulin Resistant Type 1 Diabetic Rats

**DOI:** 10.1038/srep26379

**Published:** 2016-05-20

**Authors:** Michelle S. Dotzert, Michael R. Murray, Matthew W. McDonald, T. Dylan Olver, Thomas J. Velenosi, Anzel Hennop, Earl G. Noble, Brad L. Urquhart, C. W. James Melling

**Affiliations:** 1 Exercise Biochemistry Laboratory, School of Kinesiology, Western University, London, Ontario, Canada; 2 Neurovascular Research Laboratory, School of Kinesiology, Western University, London, Ontario, Canada; 3 Department of Physiology and Pharmacology, Schulich School of Medicine and Dentistry, Western University, London, Ontario, Canada; 4 Lawson Health Research Institute, London, Ontario, Canada; 5 Department of Medicine, Schulich School of Medicine and Dentistry, Western University, London, Ontario, Canada

## Abstract

The etiology of insulin resistance in Type 1 Diabetes (T1D) is unknown, however it affects approximately 20% of T1D patients. Intramyocellular lipids (IMCL) have been identified as a mechanism of insulin resistance. We examined skeletal muscle of T1D rats to determine if alterations in lipid metabolism were evident and whether aerobic exercise training improves IMCL and insulin resistance. To do so, 48 male Sprague-Dawley rats were divided into control (C), sedentary diabetes (D) and diabetes exercise (DX) groups. Following multiple low-dose Streptozotocin (STZ) injections (20 mg/kg), glycemia (9–15 mM) was maintained using insulin treatment. DX were treadmill trained at high intensity (~75% V0_2max_; 5days/week) for 10 weeks. The results demonstrate that D exhibited insulin resistance compared with C and DX, indicated by decreased glucose infusion rate during a hyperinsulinemic-euglycemic clamp (p < 0.05). There were no differences between C and DX, suggesting that exercise improved insulin resistance (p < 0.05). Metabolomics analysis revealed a significant shift in lipid metabolism whereby notable fatty acid metabolites (arachidonic acid, palmitic acid and several polyunsaturated fatty acids) were significantly elevated in D compared to C and DX. Based on the intermediates observed, insulin resistance in T1D is characterized by an insulin-desensitizing intramyocellular fatty acid metabolite profile that is ameliorated with exercise training.

Type 1 Diabetes (T1D) is characterized by pancreatic beta cell destruction and the inability to produce insulin and maintain glycemic control. A relatively new manifestation of diabetes, termed “Double Diabetes” has been identified, whereby insulin deficiency is coupled with the development of insulin resistance. The combination of these risk factors is associated with a greater risk of cardiovascular disease than T1D or Type 2 Diabetes (T2D) alone^1^.

While the etiology of insulin resistance in “Double Diabetes” remains to be determined, it is clear that it does not parallel other insulin resistant states (i.e. T2D or obesity), as patients with T1D often do not display associated factors such as obesity^2^. It has been postulated that insulin resistance development in T1D stems from autoimmune-related mechanisms, while others suggest that chronic hyperglycemia and the accumulation of advanced glycemic end products (AGEs) are causative factors^3^,^4^,^5^. In this respect, glucotoxicity has been shown to lead to the activation of c-Jun N-terminal kinases (JNK) and protein kinase C (PKC) leading to disturbances in the insulin signalling pathway^6^,^7^. This role for hyperglycemia and cellular glucotoxicity has gained further support, as patients with T1D with a history of poor glycemic control are more likely to develop insulin resistance^6^.

Increased intramyocellular lipid (IMCL) has also been implicated in the development of insulin resistance in T1D patients. This may also be a result of poor glycemic management, as glycosylated haemoglobin (HbA_1C_) is correlated to IMCL in T1D^8^. Others have also shown a positive relationship between insulin resistance and IMCL accumulation^9^,^10^. Similar to T2D, lipotoxicity may play a role in the development of insulin resistance in T1D. In Zucker diabetic fatty rats, insufficient insulin leads to “metabolic overload” where mitochondrial function does not meet increased lipid flux^11^. In this case, free fatty acids may be converted to diacylglycerol (DAG) or metabolized into ceramides; both of which has been shown to interfere with insulin signalling^12^,^13^.

We have established a rodent model of T1D using multiple low-dose streptozotocin (STZ)-treatment and insulin therapy to replicate poorly managed glycemic control observed in clinical T1D^14^. In previous work, we have observed impairments in GLUT4 protein expression and the development of insulin resistance, which were improved with high intensity aerobic exercise training^14^,^15^,^16^. Concomitant with increases in insulin sensitivity we have shown that exercise training leads to reductions in blood lipid levels, decreased body mass, and decreased adipose tissue mass in comparison to sedentary T1D animals^15^,^17^. The purpose of this study was to determine whether insulin resistance development in a poorly controlled rodent model of T1D is associated with disturbances in lipid metabolism; and whether aerobic exercise-mediated improvements in insulin sensitivity are accompanied by changes in lipid metabolism. We hypothesised that several insulin-desensitizing lipid metabolites would be elevated in the skeletal muscle of insulin resistant T1D animals, indicative of impaired lipid metabolism, and that exercise training would restore insulin sensitivity and skeletal muscle lipid metabolism.

## Results

### Animal Characteristics

Animal weights and blood glucose concentrations are shown for pre- and post-exercise training (Table 1). These values were obtained prior to the onset of training and again upon the completion of 10 weeks of training. D and DX animals had significantly higher blood glucose pre- and post-training. Body mass was significantly lower in DX compared to C and D pre- and post-training.

### Hyperinsulinemic Euglycemic Clamp

**Figure 1:**
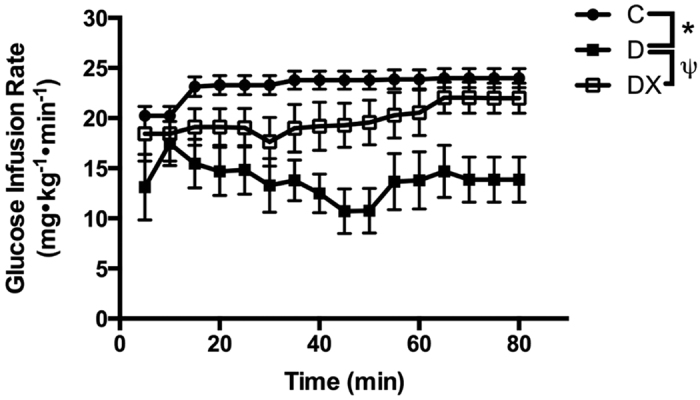
Hyperinsulinemic-euglycemic clamp. *Diabetes sedentary (D) significantly different from control. ^ψ^Diabetes exercise (DX) significantly different from (D). (C, n = 7; D, n = 8; DX, n = 9). Data are expressed as mean ± SE for each group.

Glucose infusion rates were significantly lower in D compared to C (p < 0.05) and in D compared to DX (p < 0.05), indicative of insulin resistance in the sedentary diabetic animals (Fig. 1). The glucose infusion rate was not different between C and DX (p > 0.05), indicative of an exercise-mediated improvement in insulin sensitivity.

### Metabolomics

**Figure 2:**
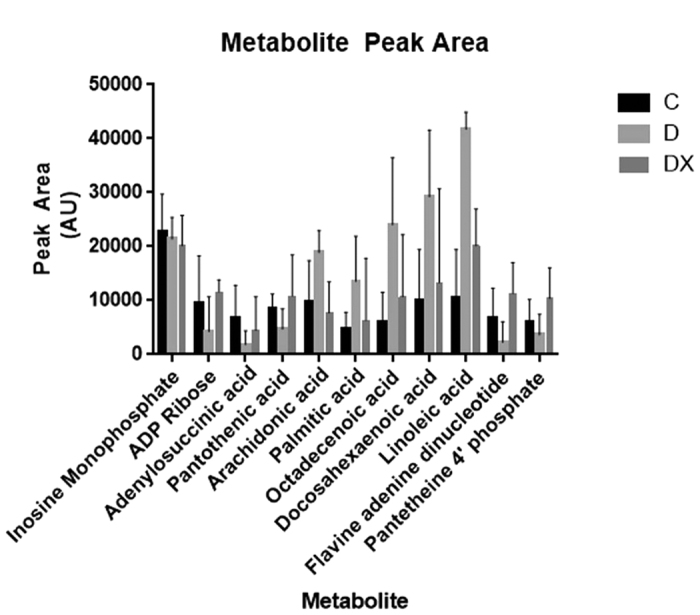
Metabolite peak area. Data represent average chromatogram peak area ± SE for each identified metabolite.

**Figure 3:**
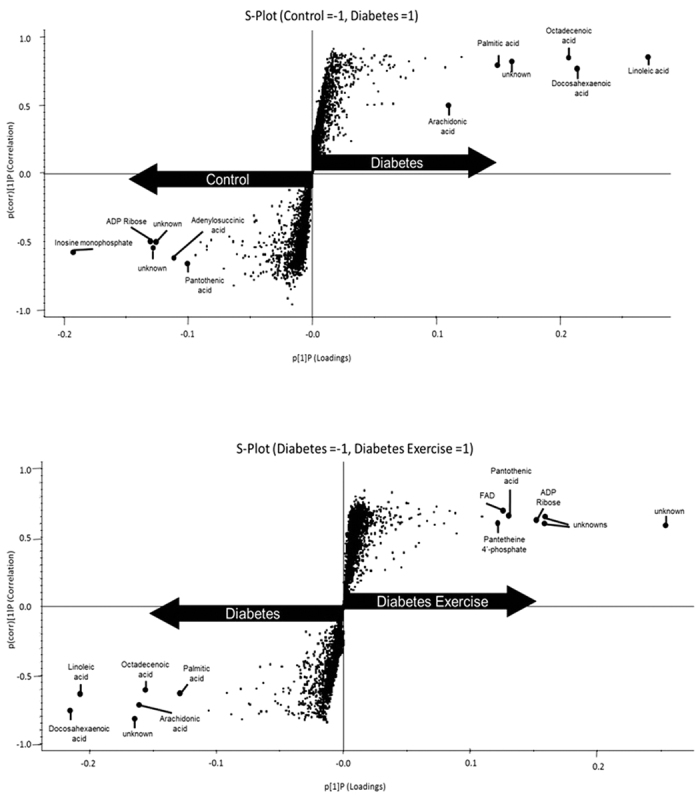
Untargeted metabolomics in the red portion of the gastrocnemius. S-plot comparison of control (C, n = 5) and sedentary diabetic (D, n = 5) (top). S-plot comparison of (D, n = 5) and diabetes exercise (DX, n = 5) (bottom). The S-plot is a visual method for identification of biomarkers. Variables farthest from the origin in the plot are deemed significant markers. Each biomarker is identified with the elemental composition from the accurate mass and comparison to fragmentation patterns from metabolite databases.

Metabolites in control and diabetes groups were described by OPLS-DA (R^2^(Y) = 0.90) with high predictive ability (Q^2^(Y) = 0.58). Those in D and DX groups were also described by OPLS-DA (R^2^(Y) = 0.92) with high predictive ability (Q^2^(Y) = 0.51). Analysis of the red portion of the gastrocnemius muscle revealed a significant increase in octadecenoic acid, palmitic acid, linoleic acid, arachidonic acid and docosahexaenoic acid in D compared to C. C demonstrated greater levels of adenosine diphosphate ribose (ADP Ribose), adenylosuccinic acid and pantothenic acid compared to D. Octadecenoic acid, linoleic acid, arachidonic acid, docosahexaenoic acid and palmitic acid also demonstrated the largest factor of change in the D compared to DX groups. Significant increases in flavine adenine dinucleotide (FAD), pantetheine 4′-phosphate, pantothenic acid and ADP ribose were observed for DX compared to D. Certain metabolites were not identifiable (designated “unknown” in figures) likely due to poor fragmentation. (Figs 2 and 3) (Table 2).

### Oil Red O

**Figure 4:**
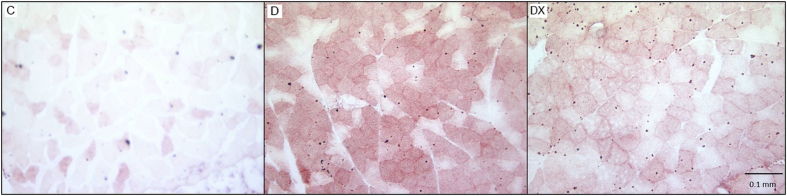
Oil Red O. Representative images of ORO staining in red vastus of sedentary control (C), sedentary diabetes (D) and diabetes exercise (DX) groups.

ORO staining for neutral lipids revealed darker stained fibres in skeletal muscle of the D group. The C group showed minimal staining, only visible in a small number of fibers. The DX group also shows minimal staining of fibers compared to the D group, but there appears to be more lipid accumulation compared to C. (Fig. 4).

### Diacylglycerol Content

**Figure 5:**
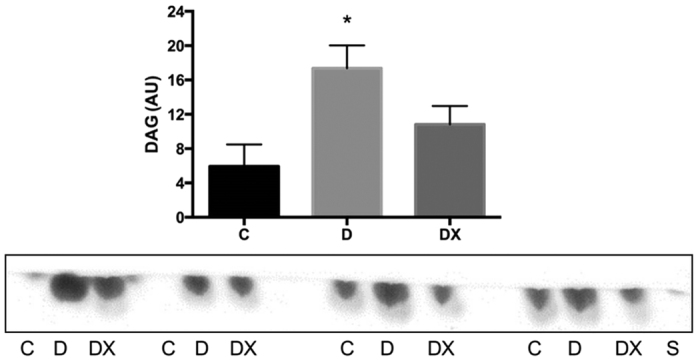
Thin layer chromatography. Quantification of DAG content in red tibialis anterior muscle in sedentary control (C, n = 4) compared to sedentary diabetic (D, n = 4) and diabetes exercise (DX, n = 4). The D group demonstrated significantly greater DAG content compared to C *(p < 0.05). A representative chromatogram run under the same experimental conditions (see methods) is shown. The representative chromatogram has been cropped to show molecule of interest. Data are expressed as mean ± SE for each group.

Thin layer chromatography of the red portion of tibialis anterior muscle revealed significantly greater DAG content in D compared to C (p < 0.05). DX did not significantly differ from C (p > 0.05), suggesting exercise improved skeletal muscle DAG content (Fig. 5).

### Hormone Sensitive Lipase Content

**Figure 6:**
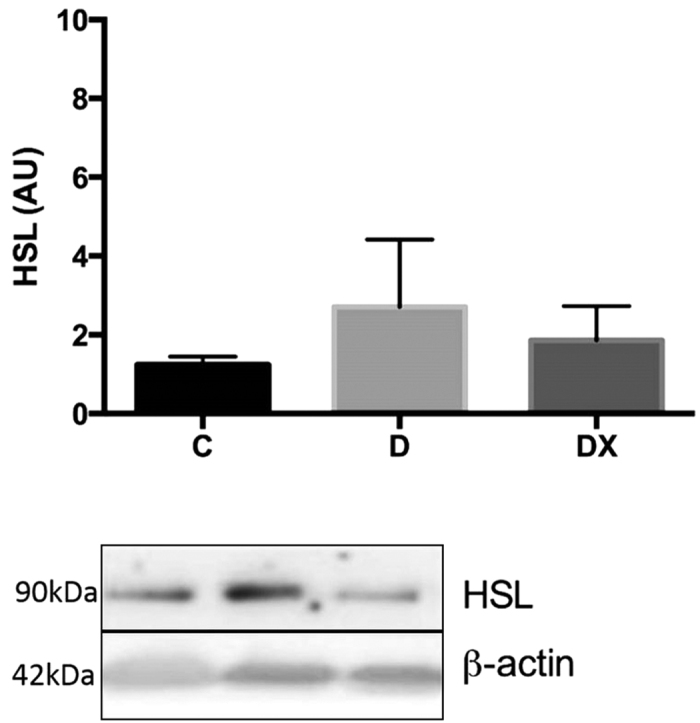
Hormone Sensitive Lipase. Total soleus HSL protein content in sedentary control (C, n = 6), sedentary diabetic (D, n = 7), and diabetes exercise (DX, n = 6). A representative blot run under the same experimental conditions (see methods) is shown. The representative blot has been cropped to show proteins of interest. There were no significant differences between groups (p = 0.556). Data are expressed as mean ± SE for each group.

We quantified HSL protein to determine whether free fatty acid accumulation was a result of greater DAG and triacylglycerol (TAG) hydrolysis. No significant differences were observed for HSL protein content of the soleus muscle between groups (p = 0.556) (Fig. 6).

## Discussion

In the current study, we were able to annotate and identify multiple metabolites that differentiate skeletal muscle from insulin resistant D animals from that of control animals. Secondly, following aerobic exercise training, DX animals showed improvements in insulin sensitivity as well as changes in metabolite levels such that they closely approximated those of healthy controls. Metabolites differentiating the D group from C and DX include arachidonic acid and palmitic acid. Given the involvement of these intermediates in inflammatory cytokine production and decreased GLUT4 translocation, these data suggest that insulin resistance in T1D elicits a shift toward the intramyocellular accumulation of insulin desensitizing, pro-inflammatory metabolites.

Arachidonic acid (AA) is a precursor to multiple inflammatory cytokines, many of which have been directly linked to the development of insulin resistance^18^. It has been reported that reductions in phospholipid membrane AA following ω-3 feeding in rats reduces the systemic inflammatory response induced by TNF-α^19^. TNF-α is responsible for the transcriptional suppression of genes relating to skeletal muscle glucose uptake including GLUT4 and peroxisome proliferator-activated receptor gamma coactivator 1 alpha (PGC-1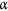) and can directly influence insulin signaling^20^. Palmitic acid, a saturated fatty acid (SFA), also impairs insulin signalling and is correlated to development of insulin resistance^21^. Palmitic acid, as well as other saturated fatty acids (SFAs), impair the insulin signal at many points, including reducing IRS-1 (insulin receptor substrate 1) and protein kinase B (PKB/Akt) phosphorylation^22^. Lastly, linoleic acid, a polyunsaturated fatty acid (PUFA), also differentiated our D from C and DX groups. Linoleic acid has been shown to contribute to insulin resistance by reducing GLUT4 protein expression in L6 muscle cells^23^. Previously, we demonstrated that reductions in insulin sensitivity in T1D rats were coupled to changes in GLUT4 protein expression, which were normalized along with insulin sensitivity following six weeks of aerobic exercise training^15^. In line with these results, the current study demonstrates that, concomitant with improvements in insulin sensitivity, excess accumulation of palmitic acid, arachidonic acid, and linoleic acid were not evident in exercised-trained diabetic animals. This suggests that aerobic exercise training can ameliorate the insulin desensitizing effects of SFAs and PUFAs, possibly through a reduction in total skeletal muscle free fatty acid content^24^.

Skeletal muscle from sedentary diabetic animals displayed greater levels of docosahexaenoic acid (DHA) accumulation. DHA has been shown to decrease the transport efficiency of the sarcoplasmic reticulum (SR) Ca^2+^-ATPase (SERCA) pump, through increased calcium (Ca^2+^) leak. It is believed that DHA causes futile SERCA pumping thereby increasing the energy requirements to transport Ca^2+^ into the SR^25^, as SR Ca^2+^ pumps account for 40–50% of resting metabolic rate in mouse skeletal muscle^26^. Indeed, Ca^2+^-ATPase activity and Ca^2+^ uptake has been shown to be elevated in cardiac and skeletal muscle in experimental T1D animals. The resultant increased intracellular Ca^2+^ is believed to play a role in the paradoxical increase in cardiac resistance to ischemia-reperfusion injury in T1D animals^16^,^27^, while elevated intracellular Ca^2+^ may increase fatigability of skeletal muscle^29^. Here, we observe that the increase in skeletal muscle DHA levels in diabetic animals is normalized following exercise training. While it cannot be ascertained whether a reduction in DHA is indicative of improved Ca^2+^-handling efficiency in exercised diabetic animals, a reduction in Ca^2+^-ATPase activity and preserved Ca^2+^ uptake has been reported following high-intensity exercise training in skeletal muscle of T1D patients^30^.

In addition to lowered DHA levels, exercised diabetic animals exhibited elevated levels of ADP ribose in comparison to sedentary diabetic animals. ADP ribose is a Ca^2+^ mobilizing metabolite that has been shown to modulate the release of Ca^2+^ from the SR in skeletal muscle^31^. While it has been shown that ADP-ribose does not reduce Ca^2 + ^leak at the ryanodine receptor levels, it may improve Ca^2+^ handling efficiency as ADP-ribose accelerates cytosolic Ca^2+^ clearance via SERCA activity^32^. Elevated ADP ribose accumulation in exercised diabetic animals may be indicative of other alterations in Ca^2+^ mediated process involving glucose uptake and metabolism^33^. For instance, ADP ribose formation is the primary signaling factor responsible for exercise mediated GLUT4 glucose uptake in high fat fed insulin-resistant mice. The increase in ADP ribose observed in the present study is likely indicative of exercise-mediated enhancement of GLUT4 translocation and improved glucose uptake as opposed to insulin mediated GLUT4 translocation^34^.

Cyclic ADP ribose is generated by ROS-mediated activation of poly ADP ribose polymerase (PARP), which splits NAD^+^ into nicotinic acid and ADP ribose^35^,^36^. Mitochondrial ROS-mediated activation of PARP contributes to ADP ribose polymers accumulating on glyceraldehyde 3-phosphate dehydrogenase (GAPDH), impeding the flow of glycolysis and activation of pro-apoptotic factors^36^. While this would seem contrary to the known benefits associated with exercise, it is plausible that excess lipid accumulation in diabetic animals may increase the pressure head point of entry into the mitochondria, elevating ROS production, and impairment of glucose metabolism via ADP ribose accumulation on GAPDH. It is yet to be determined how modifications of GAPDH and other nuclear proteins may impact skeletal muscle; however, it may reflect an increase in the normal inflammatory process used to remove partially damaged muscle cells resulting from higher levels of exercise^37^.

Adenylosuccinic acid and inosine monophosphate (IMP) differentiated the C and D groups, suggesting altered purine metabolism in T1D. Purine nucleotides act as key regulators of cell metabolism, serving as essential carriers of chemical energy such as ATP. Purine metabolite state modulates the AMP/ATP ratio and can impact mitochondrial function as well as AMPK activity which affects key cellular functions such as skeletal muscle oxidative capacity and glucose oxidation, hepatic glucose output, and glucose sensitivity^38^. Similarly, the elevation in pantothenic acid, a precursor to coenzyme A, would suggest that T1D led to a decrease in this cofactor critical for fatty acid metabolism. Pantothenic acid did not differentiate DX from C, suggesting a restoration of aerobic metabolism with aerobic exercise training. In this respect, flavin adenine dinucleotide (FAD), pantetheine 4′-phosphate, ADP ribose and pantothenic acid differentiate DX from D; indicative of improvements in oxidative pathways in our model of T1D. Enhanced free fatty acid oxidation could reduce lipid esterification as well as ceramide and DAG formation, preventing the inhibitory effects of these lipid species on the insulin signaling cascade, restoring insulin sensitivity.

While these findings identify key markers that dissociate control from diabetic animals, the mechanism underlying these differences in lipid metabolites remains unclear. It is plausible that a greater uptake of free fatty acids with insufficient oxidation may account for the observed changes^39^. Hyperglycemia may also drive IMCL accumulation as CPT-1 and beta-oxidation are inhibited in this state^40^. Moreover, circulating insulin hinders TAG and DAG hydrolysis and stimulates fatty acid esterification, potentially contributing to these changes^41^,^42^. In fact, we have previously shown significant reductions in the insulin requirement of aerobically trained animals compared to sedentary diabetic animals^16^. Studies have shown impaired insulin-stimulated glucose transport and increased ceramide, DAG and TAG in skeletal muscle incubated in palmitate^43^. Interestingly, a single bout of prior exercise protected the insulin signal and some redistribution of free fatty acids toward TAGs was observed^43^. Here, we demonstrate an increase in DAG content in the red oxidative skeletal muscle of the D group, which was normalized with aerobic exercise training. It has been shown that DAGs interfere with the insulin signal via PKCθ, and accumulation is associated with insulin resistance^13^,^44^. Similarly, exercise has been shown to preserve the insulin signal and redistribute free fatty acids toward TAG storage as opposed to ceramides, which act at the level of PKCζ^43^. Our results align with other studies, showing that DAG, but not ceramide, is more readily altered following lipid infusion^44^,^45^.

Given the elevation in DAG, we examined the expression of hormone sensitive lipase (HSL), an enzyme that displays a high DAG substrate specificity and is considered the major DAG hydrolase in several tissues^46^. Reduced HSL protein content could potentially account for the increased DAG content observed in muscle, and this metabolite has been shown to be decreased in skeletal muscle of obese, T2D individuals^47^. Our findings reveal no significant differences in HSL protein content in soleus muscle between groups. This finding is in accordance with others who have found no significant effects of exercise training on skeletal muscle HSL content^48^,^49^. It is important to note that diabetes-related changes in HSL function may be due to posttranslational regulation, as endurance training increases phosphorylation but not total protein content^50^. However, it has also been shown that reductions in HSL phosphorylation of obese subjects is entirely due to lower HSL protein content, suggesting that muscle HSL content could be representative of enzyme activity^51^. Further work is required to ascertain the mechanisms involved in the accumulation of DAG in skeletal muscle of T1D animals and the molecular means by which exercise may reduce the accumulated levels of this lipid intermediate.

Possible limitations to this study should be addressed. First, due to poor fragmentation, we were unable to identify various ceramide species. In addition to DAG, ceramide may also play an important role insulin signaling impairment, as has been well documented to occur in T2D^52^. Secondly, while Oil Red O staining provides a visual representation of neutral lipid content within the muscle, it is unable to detect polar lipids such as ceramides or phospholipids. This data serves solely as a representative image, and cannot be used to determine the differences in total intramyocellular lipid content between groups. Lastly, the measurement of food intake between groups was not examined in the current study. Considering the impact of dietary behaviour may shed further light on the causative factors leading to changes in metabolite levels and metabolic rate in diabetic animals. While hyperphagia has been well documented STZ-T1D animals, it has been reported that hyperphagia is restored and/or prevented in models of T1D following insulin treatment^53^,^54^.

In summary, our results demonstrate that moderately hyperglycemic T1D rats develop insulin resistance which is accompanied by significant alterations in skeletal muscle lipid metabolism. Ten weeks of aerobic exercise training was able to improve insulin sensitivity and ameliorate the accumulation of harmful insulin desensitizing lipid intermediates in red oxidative skeletal muscle of T1D animals. The underlying mechanisms by which exercise training improves skeletal muscle lipid metabolism and insulin receptor function in T1D need to be determined.

## Methods

### Ethics Approval and Animals

Eight-week-old male Sprague-Dawley rats (n = 48) were obtained from Charles River Laboratories, and housed two per cage at constant temperature and humidity on a 12-h dark/light cycle. Rats had access to water and standard chow *ad libitum*. The experimental protocol followed the Principles of Laboratory Animal Care (US NH publication No. 83–85, revised 1985). Ethics approval was obtained through the University of Western Ontario Research Ethics Board, in accordance with Canadian Council on Animal Care guidelines.

### Experimental Groups

Animals were randomly divided into three experimental groups; non-diabetic sedentary control (C, n = 16), diabetic sedentary control (D, n = 16), and diabetic exercise (DX, n = 16). Upon completion of the training study, each group was divided into two subgroups. The first group (C, D, DX; n = 8) underwent a hyperinsulinemic-euglycemic clamp prior to sacrifice while the second group (C, D, DX; n = 8) did not undergo the clamp procedure but were sacrificed for tissue removal. The absence of the clamp procedure in these later animals was completed in order to preserve the resting metabolic status of the muscle tissue for biochemical analysis.

### Diabetes Induction

Upon arrival rats were housed for one week in order to familiarize with their surroundings. Following this period, T1D was induced with multiple low-dose STZ injections. STZ (20 mg/kg; Sigma Aldrich, Oakville, ON, Canada) was injected into the intraperitoneal cavity for five consecutive days^55^. Diabetes was confirmed by two consecutive non-fasting blood glucose readings of ≥18.0 mmol/L. Subsequently, insulin pellets (LinShin, Toronto, ON, Canada) were implanted subcutaneously. Insulin pellet/dosages were monitored and adjusted throughout the 10-week experimental study to ensure daily non-fasting blood glucose concentrations of 9–15 mmol/L.

### Exercise Training

Following the confirmation of diabetes and implantation of the insulin pellets, rats were familiarized on a motor-driven treadmill for one week prior to the onset of the exercise-training program. This consisted of 15 minutes of progressive running up to 30 m/min for five days. Once familiarized, the exercise-training program consisted of one-hour of treadmill running at 27 m/min on a 6% gradient, five days per week for 10 weeks. This intensity of exercise has been shown to elicit 70–80% of V0_2max_^56^. To maintain continuous running, rats received small blasts of compressed air on their haunches if they broke a photoelectric beam at the rear of the treadmill.

### Hyperinsulinemic-Euglycemic Clamp

To assess insulin resistance, eight animals from each group (a subgroup, C, D, DX) underwent a hyperinsulinemic-euglycemic clamp three days following the final bout of exercise training. Prior to the clamp procedure animals were fasted for twelve hours and anaesthetized using isoflurane and an intraperitoneal injection of urethane (25 mg/kg)/α-chloralose (4 mg/kg)^57^,^58^. Once an analgesic plane was confirmed, isoflurane was removed and the urethane/α-chloralose mixture maintained the anaesthesia. A catheter was surgically inserted into the right jugular vein for insulin and glucose infusion. Insulin (Eli Lilly, Toronto, ON, CAN) was infused at 10 mU/kg/min; 0.4 μIU/mL. Glucose (EMD Millipore, Darmstadt, HE, Germany) was infused at 20 mg/kg/min, 0.2 g/mL to maintain the blood glucose concentration, based on glucose measures every 5 minutes until minute 20 and every 10 min thereafter.

### Tissue Collection

As noted above, eight animals in each group were euthanized for tissue collection three days following the final bout of exercise by isoflurane anaesthesia followed by exsanguination and cardiac excision. Due to tissue constraints, red portions of the gastrocnemius muscle were flash frozen for metabolome analysis, red portions of the vastus muscles were mounted and flash frozen for oil red O staining, soleus (primarily red) muscles were flash frozen for western blotting and the red portion of the tibialis anterior was flash frozen for thin layer chromatography.

### Skeletal Muscle Metabolomics

100 ± 3 mg of the red portion of the vastus muscle tissue was homogenized in 250 μL of ice cold HPLC grade acetonitrile containing isatin (5 μg/mL) and flurazepam (25 ng/mL) as internal standards for 2 minutes in an ice bath. Samples were vortexed, and centrifuged at 14000 rpm at 4 °C for 5 minutes. 120 μL of supernatant was removed and diluted with 30 μL of ultrapure water, for a final sample containing 80% acetonitrile. A control injection was generated by creating a pooled sample consisting of an equal volume of all injected samples. Samples were transferred to vials and 1 μL was injected in triplicate from each vial. Injections were randomized to reduce error and pooled control samples were run every six injections. Chromatographic separation was performed on a Waters Acquity Ultra Performance Liquid Chromatograph system with separation achieved using an Acquity UPLC HSS T3 column (1.8 μm particle size, 100 mm × 2.1 mm). Column temperature was maintained at 45 °C in a Waters ACQUITY UPLC I-Class system (Waters, Milford, MA). The mobile flow was set to 0.45 ml/min and consisted of water (A) and acetonitrile (B), both containing 0.1% formic acid. UPLC conditions were as follows: 0–2 mins, 1–60% B; 2–6 mins 60–85% B; 6–8 mins 85–99% B; 8–10 mins 99–1% B. A Waters Xevo^TM^ G2S-QTofMS was used for mass spectrometry, and metabolites were measured in positive and negative electrospray ionization mode. Capillary voltage and cone voltage were set at 2 kV and 40 V respectively, and the source temperature was 150 °C. Desolvation gas flow was set to 1200 L/h at 600 °C and the cone gas flow was 50 L/h. The data were acquired in centroid mode using an MS^E^ method with an m/z range of 50–1200. Leucine-enkephalin (500 ng/mL) was used as the lockmass set at a flow rate of 10 μL/min, measured every 10 seconds and averaged over 3 scans.

### Histochemistry

Oil Red O staining for neutral lipids was performed on frozen sections of the red portion of the vastus muscle. Stock solution was prepared by combining 2.5 g of Oil Red O (Sigma Aldrich, Canada) and 400 ml isopropyl alcohol (99%) and stirred for 2 h. Working solution was prepared by mixing 1.5 parts stock with 1 part ddH2O which was cooled and filtered. Slides were mounted in aqueous mounting media (10% PBS and 90% glycerol) and photographed using a Zeiss Axioskop Optical Microscope and Northern Eclipse software.

### Thin Layer Chromatography

Total lipid extraction was performed on the red portion of tibialis anterior muscle. 200–300 mg of minced tissue was submerged in chloroform:methanol (2:1, v:v) and placed in the dark for 1 h. The extract was then poured over Whatman filter paper into a glass tube. The extraction vessel was then rinsed and vortexed with 1 mL of chloroform:methanol solution, filtered and added to the original extract. Samples were dried under a steady air stream in a 30–40 °C water bath for approximately 1 hour. The extract was weighed and diluted in 50 μL chloroform:methanol (2:1).

Lipid extracts, diacylglycerol (DAG) (1-Palmitoyl-2-Oleoyl-sn-Glycerol) Avanti Polar Lipids Inc. Alabama USA) standard were run on glass plates with a silica gel matrix (Analtech TLC uniplates, Sigma Aldrich, Canada) with a mobile phase consisting of toluene:methanol (7:3, v:v). Plates were visualized using Iodine ACS Reagent (Sigma Aldrich, Canada) in a closed glass chamber. Images were obtained using a flatbed scanner and analyzed using ImageJ.

### Western Blotting

Soleus muscle from each group was homogenized in a 1:10 (w:v) ratio of homogenizing buffer (100 mmol/L NaCl, 50 mmol/L Tris base, 0.1 mmol/L EDTA, 0.1 mmol/L EGTA and 1% Tritonx100, pH 7.5), and a Bradford assay was used to determine total protein content. Polyacrylamide gels were composed of 10% acrylamide separating gel and 4% acrylamide stacking gel. Membranes were blocked in 5% non-fat dry milk and Tris buffered saline (TBS), then incubated overnight at 4 °C in anti-hormone sensitive lipase (HSL) antibody (1:1000) (ab45422, Abcam, Cambridge MA, USA). Following secondary antibody incubation (BioRad goat-anti-rabbit IgG (H + L)- HRP conjugated 1662408, and goat-anti-mouse IgG (H + L)-HRP conjugated 1721101 as per manufactures instructions), membranes were washed and visualized using a luminol-based chemiluminescent substrate (BioRad Western C Enhanced Chemiluminescent Kit, 170-5070) on a BioRad Chemidoc XRS imager. Densities were determined using Quantity One software.

### Data Analysis

Multivariate analysis of LC-MS data was achieved with Waters Markerlynx with EZinfo 2.0 (Umetrics, Umeå, Sweden) software packages. Following normalization to total marker intensity in Markerlynx, peak intensities were transferred to EZinfo. Pareto scaling dampened the selection of features with the highest variance. Principle component analysis (PCA) and orthogonal partial least squares discriminant analysis (OPLS-DA) was performed between control and diabetic groups, and diabetic and diabetic exercise groups using EZinfo. Metabolites were identified using METLIN (http://metlin.scripps.edu) and HMDB (http://www.hmdb.ca/) databases. Fragmentation patterns for each metabolite were compared to putative database compound fragmentation using MassFragment®.

For Western Blots, hyperinsulinemic-euglycemic clamp and thin layer chromatography, group differences were tested using a one-way ANOVA and Tukey’s post hoc test with a p value at p < 0.05.

## Additional Information

**How to cite this article**: Dotzert, M. S. *et al*. Metabolomic Response of Skeletal Muscle to Aerobic Exercise Training in Insulin Resistant Type 1 Diabetic Rats. *Sci. Rep.* **6**, 26379; doi: 10.1038/srep26379 (2016).

## Figures and Tables

**Table 1:** Animal characteristics. Blood glucose concentration and body mass from pre- and post-training are represented as mean ± SE.

**Group**	**Pre-Training**		**Post-Training**	
	**Blood Glucose (mM)**	**Mass (g)**	**Blood Glucose (mM)**	**Mass (g)**
Control	4.6 ± 0.1	385 ± 6.4	4.2 ± 0.1	550 ± 11.7
Diabetic	10.2 ± 1.2*	304 ± 5.4*	16.2 ± 0.8*	460 ± 7.9*
Exercise-trained Diabetic	9.4 ± 1.2*	292 ± 7.9*^,°^	16.9 ± 0.5*	415 ± 12.1*^,°^

(*indicates a main effect for diabetes (p < 0.05), °indicates a main effect of exercise (p < 0.05)).

**Table 2:** Metabolomic analysis. Metabolites that largely differentiate control (C), sedentary diabetics (D) and exercise-trained diabetics (DX) from the red portion of the gastrocnemius are presented.

**Identity**	**Empirical Formula**	**Mass (m/z)**	**t**_**R**_**(min)**	**P[1]P**	**p(corr)[1]P**	**S-plot VIP score**	**Fold change**	**Effect**	**ID level**
Inosine monophosphate	C10H13N4O8P	347.0477	0.7	−0.190376	−0.593433	12.6789	5.1	↑C vs D	2
ADP Ribose	C15H23N5O14P2	558.0647	0.65	−0.102561	−0.501781	7.15977	2.8	↑C vs D	2
Adenylosuccinic acid	C14H18N5O11P	462.0664	1.07	−0.114934	−0.612991	7.96882	7.8	↑C vs D	2
Pantothenic acid	C9H17NO5	218.1026	1.23	−0.103502	−0.65815	7.64157	2.0	↑C vs D	2
Arachidonic acid	C20H32O2	303.2326	6.68	0.111519	0.501134	7.64936	1.6	↑D vs C	2
				−0.165695	−0.716968		2.4	↑D vs DX	
Palmitic acid	C16H32O2	255.2323	7.42	0.152801	0.800222	10.7176	2.6	↑D vs C	1
				−0.131925	−0.634719		2.2	↑D vs DX	
Octadecenoic acid	C18H34O2	281.248	7.63	0.215689	0.844199	14.9743	3.6	↑D vs C	2
			−0.165018	−0.603556		2.2	↑D vs DX		
Docosahexaenoic acid	C22H32O2	327.2325	6.5	0.221078	0.773611	16.1763	2.7	↑D vs C	2
Linoleic acid	C18H32O2	279.2322	6.82	0.279119	0.858839	19.7256	3.3	↑D vs C	2
				−0.216401	−0.648384		2.0	↑D vs DX	
Flavine adenine dinucleotide (FAD)	C27H33P2N9O15	784.1504	1.33	0.133381	0.677607	6.84121	6.7	↑DX vs D	2
Pantetheine 4′-phosphate	C11H23N2O7PS	357.0886	1.23	0.125091	0.597866	1.22198	3.0	↑DX vs D	2
Unknown	–	347.0394	0.97	−0.140527	−0.529978	10.0537	3.2	↑ C vs D	2
				0.175433	0.646263		3.7	↑ DX vs D	
Unknown	–	558.0647	0.65	−0.102561	−0.501781	7.15977	2.8	↑ C vs D	2
Unknown	–	329.2481	6.88	−0.16972	−0.826997	11.8857	3.4	↑ D vs C	2
				−0.16972	−0.826997		3.3	↑ D vs DX	
Unknown	–	347.0392	0.7	0.257514	0.590923	0.377651	1.9	↑ DX vs D	2
Unknown	–	695.0872	1.23	0.154213	0.604018	1.221986	2.2	↑ DX vs D	2
